# What Is Success? A Narrative Review of Research Evaluating Outcomes of Antibiotics Used for Treatment of Clinical Mastitis

**DOI:** 10.3389/fvets.2021.639641

**Published:** 2021-02-02

**Authors:** Pamela L. Ruegg

**Affiliations:** David J. Ellis Chair in Antimicrobial Resistance & Professor of Large Animal Clinical Sciences, College of Veterinary Medicine, Michigan State University, East Lansing, MI, United States

**Keywords:** dairy, antibiotics, bacteria, veterinary, mastitis

## Abstract

Treatment of clinical mastitis is the most common reason that antimicrobials are given to adult dairy cows and careful consideration of treatment protocols is necessary to ensure responsible antimicrobial stewardship. Clinical mastitis is caused by a variety of bacteria which stimulate an immune response that often results in spontaneous bacteriological clearance but can develop into long-term subclinical infections. Use of antimicrobial therapy is most beneficial for cases that are caused by pathogens that have a low rate of spontaneous cure but high rate of therapeutic cure. The purpose of this paper is to review studies that evaluated outcomes of antimicrobial therapy of clinical mastitis. Few studies reported differences in bacteriological cure among treatments and this outcome was rarely associated with clinical outcomes. Return to normal milk appearance was evaluated in most studies but demonstrated little variation and is not a reliable indicator of therapeutic success. Somatic cell count should be measured at quarter-level and will decline gradually after bacteriological clearance. Few researchers have evaluated important clinical outcomes such as post-treatment milk yield or culling. Few differences among approved antimicrobial therapies have been demonstrated and selection of antimicrobial therapy should consider the spectrum of activity relative to etiology.

## Introduction

Mastitis is the most common bacterial disease of mature dairy cows (1–3) and is diagnosed based on recognition of an inflammatory response initiated after the immune system detects intramammary infection (IMI). Like most bacterial diseases, the magnitude of the inflammatory response is dependent on virulence of the pathogen and is regulated by the ability of the host to mount a rapid and effective immune response (4). A mild inflammatory response results in an influx of neutrophils into the gland without any visible changes in the gland (subclinical disease) whereas a larger inflammatory response results in observable localized or generalized signs (clinical mastitis). Subclinical mastitis is the most common outcome of IMI and is defined by enumeration of somatic cells in milk. When somatic cell count (SCC) of milk exceeds a healthy threshold (usually > 150,000 or 200,000 cells/mL) (5) the gland is considered to be subclinically infected. Milk of cows affected with subclinical mastitis has a normal appearance and can be co-mingled with milk from healthy cows and sold for consumption, so treatment of subclinical mastitis during lactation is rarely recommended (6, 7).

Clinical mastitis is diagnosed when the magnitude of the inflammatory response is sufficient to result in visible changes in the milk, the mammary gland or the cow. The incidence of clinical mastitis is estimated to range between about 17–40 cases per 100 cows per year (8–12). Clinical mastitis is often assessed based on severity of the presenting signs and only a small proportion of cows have acute systemic disease that requires immediate therapy (13–16). The appearance of clinical signs and the necessity of discarding abnormal milk results in strong motivation for farmers to effectively treat clinical cases (17). In the U.S., most cases of are treated with IMM products containing 1st or 3rd generation IMM cephalosporins without knowledge of etiology (8). Selection of antimicrobials was reported to be based on “historical effectiveness” (92% of farmers), “veterinary recommendation” (66%), “historical culture and sensitivity results,” (33%) or individual cow culture before treatment (22%). The definition of “historical effectiveness” was not supplied and is illustrative of the ambiguities associated with evaluation of mastitis treatments. Oliveira et al. (18) reported treatment of 589 cases of clinical mastitis occurring on 51 Wisconsin dairy farms. Most cases were treated solely with IMM antibiotics but about 30% received either a second IMM antibiotic or were treated with both IMM and systemic antibiotics. In that study, farmers collected milk samples before treatment and later submitted them to a research laboratory. The culture results demonstrated that 32% of IMM antibiotics were administered to clinical cases that were bacteriologically negative before treatment and an additional 19% of IMM antibiotics were given to cases caused by *E. coli*. In these herds, symptomatic treatment of non-severe cases of clinical mastitis without determination of etiology resulted in over-prescription of antibiotics in almost 50% of cases. More recently, treatment data was reported for >26,000 cases of clinical mastitis occurring on 40 large Wisconsin dairy herds (2, 19). Based on review of computerized health records, the incidence of clinical mastitis on these farms was 34% and use of antimicrobials varied greatly. About 31% of cases were not treated using antimicrobials, while 53% received approved products containing IMM ceftiofur, 10% received IMM cephapirin, 3% each were treated with IMM hetacillin or pirlimycin and <1% received IMM amoxicillin. Systemic antibiotics were given to 14% of cases on 29 farms. The average duration of treatment using IMM antibiotics ranged from 3.3 to 5.7 d. There was no indication that efficacy varied among treatments.

Symptomatic treatment without knowledge of etiology results in unnecessary antimicrobial treatments (such as use of antimicrobials for treatment of culture negative cases) (18) and it is impossible to determine etiology without use of diagnostic tests (such as culturing or DNA based technologies). Knowledge of etiology is fundamental to prescribe an appropriate treatment and is necessary to properly evaluate outcomes. Pathogens vary in virulence and possess differing abilities to stimulate an immune response that may result in spontaneous bacteriological cure. Differences in bacterial cell wall structures result in differing susceptibilities to antimicrobials, and most antibiotics approved for treatment of mastitis have limited ability to inhibit or destroy Gram-negative bacteria. Exposure to mastitis pathogens varies among herds but overall, the distribution of etiologies on modern dairy farms is fairly consistent. When milk samples from quarters affected with clinical mastitis are properly collected and assessed, the results are typically distributed as no growth (15–30%), Gram-negative (20–30%), gram-positive (20–25%), and 10–15% other pathogens (*Prototheca* spp, *Serratia* spp., yeasts and others) (3, 10, 12, 18, 20, 21). While there are some exceptions, it is difficult to justify the use of antimicrobial therapy for treatment of non-severe cases of clinical mastitis that are culture negative (15, 16, 22–24) or Gram-negative (25–27) and inclusion of these cases in positively controlled efficacy studies may result in over-estimating the impact of antimicrobial therapy. Thus, pathogen-specific evaluation of therapeutic outcomes is strongly recommended.

Use of culture-based protocols to guide selective therapy have been shown to be cost effective and result in more judicious antimicrobial usage (16, 24, 28) but some researchers have created economic models suggesting that delayed therapy may have negative consequences for herds that have a significant proportion of clinical mastitis caused by Gram-positive organisms (29). Recommendations for treatment are frequently based on outcomes defined in clinical trials but relatively few clinical trials have been performed to generate evidence based protocols. On farms, treatment efficacy is often judged by the speed of return to normal appearance of milk, but this outcome has little variation and is not a good indicator of therapeutic success (30) so an understanding of the importance of other outcomes is needed. The purpose of this paper is to review clinical trials that were conducted to assess antimicrobial treatments of clinical mastitis and discuss the strengths and limitations of outcomes used to evaluate therapeutic success.

## Considerations When Evaluating Mastitis Treatments

### Spectrum of Activity

When evaluating outcomes of mastitis therapy, the spectrum of activity of the antimicrobial should be considered relative to the etiology of the cases enrolled in the study. In most countries, a variety of IMM antimicrobials are approved and veterinarians in some countries have access to systemically administered drugs that are able to penetrate mammary gland barriers (21). Most approved antimicrobials are relatively narrow spectrum (target Gram-positive organisms) but in some countries, broader spectrum products such as 3rd and 4th generation cephalosporins and some quinolones are approved. Dairy farmers in the U.S. have access to 7 approved IMM antimicrobial products but no systemic products are approved for treatment of clinical mastitis (limited extralabel usage of some products is allowed). One approved IMM product is classified as a lincosamide (pirlimycin) while 6 IMM products are classified as beta-lactams. The beta-lactams include 1st (cephapirin) and 3rd (ceftiofur) generation cephalosporins, aminopenicillins (amoxicillin and hetacillin), penicillin G, and a penicillinase-resistant penicillin (cloxacillin) (31). All approved IMM products are expected to have efficacy against most common Gram-positive mastitis pathogens and most are labeled as efficacious against *Streptococci* and *Staphylococci*. While there is limited research to differentiate among products, based on the antimicrobial classes, it is unlikely that there are significant differences among approved IMM products in efficacy for treatment of common Gram-positive pathogens. Use of antimicrobials for treatment of non-severe mastitis caused by *E. coli* is generally not needed (26) because these cases have a high rate of spontaneous cure (25, 26), thus very few cases of clinical mastitis benefit from use of a broader spectrum antimicrobials.

### Intrinsic and Acquired Resistance

Before antimicrobials are approved for treatment of mastitis they are required to demonstrate efficacy for pathogens that are listed on the product label. Before antimicrobials are used to treat pathogens that are not listed on the label, the possibility of intrinsic resistance should be considered. Intrinsic resistance occurs when a bacterial genus or species lacks targets or possess defenses to render an antimicrobial ineffective. For example, Gram-negative bacteria are intrinsically resistant to pirlimycin because their cell walls lack a binding site. Most Gram-negative bacteria are intrinsically resistant to penicillin G, many *Klebsiella* spp. are intrinsically resistant to aminopencillins and intrinsic resistance to ampicillin and 1st and 2nd generation cephalosporins are common in *Enterobacter* spp. (32). Two IMM products (ceftiofur and hetacillin) have label claims that include efficacy for *E. coli*, but no products have efficacy claims for treatment of mastitis caused by *Klebsiella* spp. Some studies evaluating antimicrobial therapies of mastitis have included cases that are intrinsically resistant to the product (33, 34) and in these instances outcomes cannot be attributed to antimicrobial therapy. Knowledge of etiology is necessary to ensure that the spectrum of activity of an antimicrobial is appropriate for the case.

Acquired resistance refers to acquisition of resistance by a normally susceptible bacterial strain through some kind of genetic modification and is usually recognized by bi-modal distribution of minimum inhibitory concentration (MIC) values. Monitoring acquired resistance is useful to identify the potential of transmission of genetic determinants of resistance into environments and food systems. While there is little evidence that most mastitis pathogens found in N. American dairy herds have acquired widespread resistance to most IMM antimicrobials (35), a bimodal distribution of MIC values was observed for about one-third of *Klebsiella pneumoniae* included in a recent trial (25) and susceptibility of pathogens that are not included on product labels should be monitored on a regular basis.

### Accounting for Cow Factors

Therapeutic success is driven by both pathogen factors and cow characteristics (30, 36–38). Effective bacterial clearance depends on a robust immune response and factors such as parity, stage of lactation, and history of previous clinical or subclinical mastitis cases should be considered when evaluating efficacy of treatments administered for clinical mastitis.

## Methods Used to Evaluate Research About Clinical Mastitis Treatment

### Inclusion of Trials in This Review

Studies included in this review were retrieved by searching databases and web platforms using PubMed, Web of Science, and Google Scholar. Addition studies were added by reviewing bibliographies of relevant papers. Boolean search terms were used and included mastitis, bovine, clinical, randomized, non-inferiority and treatment. Only natural exposure clinical trials that utilized randomized or systematic allocation to evaluate antimicrobial treatments and were published since 2000 in English language journals were retrieved. Only studies from the last 20 years were included because there have been considerable changes in the distribution of pathogen from contagious organisms (such as *S. aureus* and *S. agalactiae*), to a more diverse mix of environmental organisms (5), use of antimicrobials is increasingly discouraged and management of clinical mastitis has gradually shifted to selective therapy of clinical cases using on-farm culture.

Studies that did not define the antimicrobial therapy or included only non-antimicrobial therapies were excluded. Studies evaluating homeopathic or herbal treatments were also excluded. While thorough, the search was not systematic and although no studies that met inclusion criteria were excluded, it is likely that some qualifying studies were missed.

Despite the global importance of bovine mastitis, relatively few clinical trials that evaluated antimicrobial treatments of clinical mastitis were identified (Table 1). While a systematic review or meta-analysis is the ideal method to summarize and compare studies, the wide diversity of study designs, variation in outcomes, differences in pathogens and treatment protocols included in mastitis trials creates a challenging situation relative to use of this method. The limited number of trials evaluating antimicrobial therapy used for treatment of bovine mastitis has been previously noted by authors of a systematic review who were unable to identify sufficient papers to establish networks to evaluate bacteriological cure and were unable to reach a conclusion about clinical efficacy of antimicrobials (59). Studies included in this review were conducted in the U.S. or Canada (*n* = 9), European Union (*n* = 8), New Zealand (*n* = 6), Brazil (*n* = 2) and Mexico (*n* = 1). The 26 studies included 65 study arms that included IMM therapy containing a single antimicrobial (*n* = 28), IMM therapy containing combination products [*n* (60) = 13], combined IMM and systemic therapy (*n* = 5), systemic therapy only (*n* = 11), non-antibiotic treatments (*n* = 2) and no treatment (negative control; *n* = 6). Antibiotic classes included penicillins and extended spectrum penicillins, penicillin and aminoglycoside combinations, 1st−4th generation cephalosporins, lincosamides, a lincosamide combined with an aminoglycoside, tetracycline combined with several other classes, fluoroquinolones and macrolides. No studies were replicated, and a variety of outcomes were reported.

**Table 1:** Description of 26 clinical mastitis treatment trials published since 2000 and meeting inclusion criteria for this review.

**Citation, Location & Cases (n)**	**Approximate distribution of pathogens^**a**^**	**Treatments evaluated**
**NEGATIVELY CONTROLLED RANDOMIZED CLINICAL TRIALS**		
Roberson et al. (39) USA, 85 cases	12% NG^b^ 40% Gram negative 33% *Streptococcus* spp. 3% *S. aureus* 12% other	1. Amoxicillin IMM for 1.5 d 2. Frequent Milk out only for 3 d @ 4 time per d 3. Amoxicillin IMM (1.5 d) + Frequent milk out 4. No antibiotic nor frequent milk out
Suojala et al. (27) Finland, 132 cases	100% *E. coli*	1. Enrofloxacin – systemic for 2 d 2. Ketoprofen – oral for 1–3 d
Schukken et al. (40) USA, 104 cases	47% *E. coli* 39% *Klebsiella* spp. 14% *Enterbacter* spp.	1. Ceftiofur IMM for 5 d 2. No treatment
Persson et al. (41) Sweden, 56 cases	100% *E. coli*	1. Enrofloxacin – Systemic for 3 d 2. No treatment
Fuenzalida and Ruegg (22) USA, 121 cases	100% NG	1. Ceftiofur IMM for 5 d 2. No treatment
Fuenzalida and Ruegg, (25) USA, 168 cases	47% *E. coli* 46% *Klebsiella pneumoniae* 8% other	1. Ceftiofur IMM for 2 d 2. Ceftiofur IMM for 8 d 3. No Rx
**POSITIVELY CONTROLLED TRIALS - NOT TESTING A NON-INFERIORITY HYPOTHESIS**		
Erskine et al. (34) USA, 104 cases (only severe cases)	20% NG 54% Gram negative 13% *Streptococcus* spp. 0% *S. aureus* 13% other	1. Ceftiofur SYS for 5 d + IMM pirlimycin for 3 d 2. IMM pirlimycin for 3 d All cows received supportive fluids at initiation of treatment and anti-inflammatories
Wraight (42) New Zealand, 416 cases	12% NG 8% Gram negative 34% *Streptococcus* spp. 18% *S. aureus* 28% other	3. Cefuroxime IMM for 1.5 d 4. Cloxacillin IMM 1 tube Q 48 h for 3 total Rx
McDougall (43) New Zealand, 404 cases	51% NG 2% Gram negative 30% *Streptococcus* spp. 5% *S. aureus* 12% other	1. Lincomycin/Neomycin IMM for 1.5 d 2. Penicillin/streptomycin IMM for 1.5 d
Taponen et al. (44) Finland, 117 cases	100% β-lactamase neg *S. aureus*	1. Penicillin G IMM for 4 d 2. Penicillin G/neomycin IMM for 4 d Both groups also received systemic PPG on day 1
Serieys et al. (45) France, 227 cases	18% NG 28% Gram negative 18% *Streptococcus* spp. 13% *S. aureus* 23% other	1. Penethemate systemic for 3 d 2. Cloxacillin/Ampicillin IMM for 3 d
Taponen et al. (46) Finland, 166 cases	100% *S. aureus*	1. B-lactamase neg: Systemic Penicillin for 5 d & IMM Penicllin/neomycin for 4d 2. B-lactamase neg: Systemic Pencillin for 5 d 3. B-lactamase pos: Systemic Amoxicillin/Clavulanic acid for 5d & IMM Amoxicillin/clav.acid/prednisolone for 4 d 4. B-lactamase pos: Sys. Spiramycin for 5 d
Wenz et al. (33) USA, 144 Cases	0% NG 68% Gram negative 15% *Streptococcus* spp. 0% *S. aureus* 31% other	1. Pirlimycin IMM for 2 d 2. Pirlimycin IMM for 2 d & systemic Ceftiofur for 3 d 3. Cephaparin IMM for 3 d (2x/d) 4. Cephaparin IMM for 3 d (2x/d) & systemic Ceftiofur for 3 d
Bradley and Green (47), EU, 491 cases	4% NG 25% Gram negative 36% *Strep sp*. 12% *S. aureus* 23% other	1. Cefalexin/Kanamycin IMM for 2 d 2. Cefoperazone IMM for 2 d 3. Cefquinome IMM for 1.5 d
Swinkels et al. (48) EU, 206 cases	100% *S. aureus*	1. Cefquinome IMM for 1.5 d 2. Cefquinome IMM for 5 d
Kalmus et al. (49) Estonia, 140 cases	0% NG 0% Gram negative 71% *Streptococcus* spp. 6% *S. aureus* 23% other	1. BenzylPenicillin systemic for 5 d 2. BenzylPenicillin IMM for 5 d
Truchetti et al. (50) Canada, 241 cases	32% NG 9% Gram negative 20% *Streptococcus* spp. 26% *S. aureus* 13% other	1. Ceftiofur IMM for 2 d 2. Ceftiofur IMM for 5 d
Cortinhas et al. (51) Brazil, 264 cases	50% NG 10% Gram negative 22% *Streptococcus* spp. 8% *S. aureus* 10% other	1. Ceftiofur IMM for 4d (moderate cases also received prednisolone) 2. Tetracycline/neomycin/bacitracin/prednisolone IMM for 4d
Viveros et al. (52) Mexico, 292 cases	38% NG 18% Gram negative 13% *Streptococcus* spp. 9% *S. aureus* 22% other	1. Enrofloxacin suspension IMM for 3 d 2. Enrofloxacin powder IMM for 3 d 3. Ceftiofur IMM for 3 d 4. Enrofloxacin systemic for 3 d
McDougall et al. (53) New Zealand, 304 cases	23% NG 4% Gram negative 55% *Streptococcus* spp. 5% *S. aureus* 13% other	1. Amoxicillin/clavulanic acid IMM for 1.5 d 2. Amoxicillin/clavulanic acid IMM for 2.5 d
**POSITIVELY CONTROLLED TRIALS – TESTING A NON-INFERIORITY OR “EQUIVALENCY” HYPOTHESIS**		
McDougall et al. (54) NZ, 1,561 cases	23% NG 1% Gram negative 38% *Streptococcus* spp. 17% *S. aureus* 18% other	1. Penicillin G IMM for 1-1.5 d 2. Cefuroxime IMM for 1-1.5 d 3. Pencillin/streptomycin IMM for 1-1.5 d
McDougall et al. (21) New Zealand, 662 cases	0% NG 0% Gram negative 79% *Streptococcus* spp. 6% *S. aureus* 15% other	1. Penethemate systemic for 3 d 2. Tylosin systemic for 3 d
Schukken et al. (55) USA, 296 cases	28% NG 25% Gram – 23% *Streptococcus* spp. 3% *S. aureus* 21% other	1. Cephaparin IMM for 1 d 2. Ceftiofur IMM for 5 d
Vasquez et al. (56) USA, 588 cases	36% NG 22% Gram negative 22% *Streptococcus* spp. 8% *S. aureus* 12% other	1. Ceftiofur IMM for 5 d 2. Hetacillin IMM for 3 d
Bryan et al. (57) New Zealand, 458 Cases	0% NG 3% Gram negative 58% *Streptococcus* spp. 27% *S. aureus* 12% other	1. Penicillin/cloxacillin IMM for 3 d 2. Tetracycline/oleandromycin/neomysin/prednisolone IMM for 3 d
Tomazi et al. (58) Brazil, 346 cases	30% NG 12% Gram negative 18% *Streptococcus* spp. 10% *S. aureus* 30% other	1. Cephaparin/prednisolone IMM for 2 d 2. Tetracycline/neomycin/bacitracin/prednisolone IMM for 2 d

a *Overall enrollment estimated from overall etiologies reported in results, subsets of data were often used to calculate various outcomes*;

b *No growth on culture*.

### Study Designs Included in This Review

Mastitis efficacy trials are challenging to perform and use different study designs. Some trials are performed using experimental challenge where cows are purposefully infected using a well-described bacterial strain (61, 62). Experimental challenge studies are useful for answering narrow research questions, but natural exposure trials are preferred for evaluating treatments and no challenge studies were included. Natural exposure trials randomly allocate cows to treatments and compare outcomes to either a non-treated control group or a positive control group. Non-treated (“negatively-controlled”) control groups are considered the gold standard for determining efficacy and are able to determine if treatments improve (“are superior to”) outcomes as compared to non-treated cows (or cows treated with a placebo). The null hypothesis in a superiority trial states that treatments are equally effective while the alternative hypothesis states that they differ. The inclusion of non-treated animals allows the determination of spontaneous cure so the additional benefit of treatment can be determined. Welfare concerns about not treating cows affected with mastitis have limited the number of negatively controlled trials that have been performed and only 6 of the 26 trials included in this review were negatively controlled. Only one of the negatively controlled studies included Gram-positive pathogens (39).

Positively controlled clinical trials are frequently used to assess mastitis treatments (Table 1) and can be designed to demonstrate superiority or non-inferiority. Of positively-controlled studies included in this review (*n* = 20), 6 were specifically designed to test non-inferiority, 1 stated that they were testing a superiority hypothesis, while the remainder (*n* = 13) compared outcomes among treatments but did not define the type of hypothesis that they were testing. The lack of a non-treated control group makes it impossible to separate treatment effects from spontaneous cures and superiority is rarely demonstrated. The null hypothesis in a non-inferiority trial states that the treatments differ while the alternative hypothesis states that they are non-inferior. Non-inferiority studies, include a pre-defined margin of non-inferiority for each outcome (usually 15%) and conclude that the new treatment is superior, non-inferior, inconclusive or inferior to the comparison treatment (55, 63). It is important to recognize that the inclusion of culture-negative cases in trials will skew the results toward positive outcomes (regardless of treatment) as the majority of these cases have achieved spontaneous bacteriological cure at the time of detection (22, 23). Mathematical realities dictate that inclusion of a large proportion of culture-negative and non-severe Gram-negative cases in non-inferiority trials will almost always result in a finding of non-inferior unless the “true-efficacy” of one of the products is very low. Of 6 non-interiority trials included in this review, 4 enrolled cases regardless of etiology (including culture-negative) and all 6 concluded that the “new treatment” was non-inferior (or inconclusive) to the comparator product. All of the non-inferiority trails included in this review evaluated commercially available products which infers that the drug approval process resulted in an acceptable level of efficacy for at least some outcomes. Outcomes of non-inferiority trials should always be evaluated relative to the distribution of pathogens enrolled in the study with emphasis on the number of enrolled cases that would likely be within the spectrum of activity of the products being compared.

## Outcomes Evaluated to Determine Efficacy

Clinical trials can include both microbiological and clinical outcomes but other than bacteriological cure (BC), there is little consistency in outcomes that are reported (Table 2). In the studies included in this review, BC was reported by 23 of 26 studies. Other outcomes include new intramammary infections (NIMI) (reported in 6 studies), clinical cure (24 studies), recurrence of another clinical case (4 studies), post-treatment SCC (14 studies), post-treatment milk yield (6 studies), culling (8 studies), and miscellaneous other outcomes (such as measures of inflammation or variations of BC). Publication bias does not seem to have influenced these trials as only half of the studies reported a significant difference among treatment groups for at least 1 outcome.

**Table 2:** Outcomes evaluated in 26 clinical mastitis treatment trials.

**Study**	**Bact. cure**	**New IMI**	**Clinical cure**	**SCC**	**Recurrence**	**Milk yield**	**Culling**
**NEGATIVELY CONTROLLED RANDOMIZED CLINICAL TRIALS**							
Roberson et al. (39)	NSD^a^	NSD	NSD	SIG^b^	Defined as CC NSD	SIG	
Suojala et al. (27)	NSD		NSD at day 21	No stats			NSD
Schukken et al. (40)	SIG		SIG	NSD		NSD	SIG
Persson et al. (41)				SIG			
Fuenzalida and Ruegg (22)		NSD	NSD	NSD	NSD	NSD	NSD
Fuenzalida and Ruegg (25)	SIG		NSD	NSD	NSD	NSD	NSD
**POSITIVELY CONTROLLED TRIALS – NOT TESTING A NON-INFERIORITY HYPOTHESIS**							
Erskine et al. (34)			NSD				
Wraight (42)	NSD		NSD				
McDougall (43)	NSD		SIG	NSD	SIG	NSD	
Taponen et al. (44)	NSD		NSD				
Sérieys et al. (45)	NSD		NSD	SIG			
Taponen et al. (46)	SIG		NSD				
Wenz et al. (33)	NSD				NSD		NSD
Bradley and Green (47)	NSD	NSD					
Swinkels et al. (48)	NSD	NSD	SIG	NSD			
Kalmus et al. (49)	NSD		NSD	NSD			NSD
Truchetti et al. (50)	SIG	NSD	NSD				
Cortinhas et al. (51)	NSD	NSD	NSD				
Viveros et al. (52)	NSD		SIG	SIG			
McDougall et al. (53)	NSD		SIG	NSD			
**POSITIVELY CONTROLLED TRIALS – TESTING A NON-INFERIORITY OR “EQUIVALENCY” HYPOTHESIS**							
McDougall et al. (54)	NSD		SIG		Defined as CC		
McDougall et al. (21)	NSD		SIG	NSD	Defined as CC	NSD	NSD
Schukken et al. (55)	NI^c^		NSD				NI
Vasquez et al. (56)	NI		NI	NI			NI
Bryan et al. (57)	NSD		NSD		Defined as CC		
Tomazi et al. (58)	Inconclusive		Inconclusive	NI			

a *No significant difference*;

b *statistically significant difference among treatments*;

c *non-inferior*.

### Bacteriological Cure and New Intramammary Infection

The purpose of antibiotic treatment is to enhance clearance of bacterial pathogens and treatment efficacy is initially evaluated based on estimates of BC. This outcome is very relevant for approving new products but is rarely evaluated in a clinical setting. Bacteriological cure is assessed by comparison of recovery of bacteria from milk samples collected when the case is detected to recovery of the same isolate from milk samples collected at various intervals after treatment is completed. Sampling strategies and intervals used to define BC vary among studies (Table 3). Some researchers defined BC based on results of a single post-treatment milk sample, while other studies require the absence of the causative pathogen in multiple samples (usually collected at 7-d intervals). In the included studies, apparent BC ranged from about 27–95%, but it is important to recognize that comparisons among studies are not accurate due to differences in the distribution of pathogens and sampling periods. The overall means and ranges of BC were 69% (27–95%; *n* = 35), 68% (33–91%; *n* = 13) and 60% (38–87%; *n* = 6) for all IMM antimicrobial therapies, systemic or systemic and IMM therapies combined or no antimicrobial treatment, respectively.

**Table 3:** Bacteriological cure (BC) definitions and outcomes for 24 studies reporting this outcome.

**Study**	**Bacteriological cure definition**	**% BC^**a**^**	**Treatment effects**
**NEGATIVELY CONTROLLED RANDOMIZED CLINICAL TRIALS**			
Roberson et al. (39)	Etiology absent 3 consecutive days at 7 & 36 d	67.0% 45.0% 53.0% 55.0%	No
Suojala et al. (27)	No *E. coli* at d 2 or d 21	90.5% 86.8%	Not at d 21
Schukken et al. (40)	Etiology absent at d 7 & 14; if samples missing failure was defined	73.0% 38.0%	Yes
Persson et al. (41)	Etiology absent at d 3 & 28	88.5% 84.2%	No statistics performed
Fuenzalida and Ruegg (25)	Etiology absent at 7, 14, 21, 28 d samples	70.3% 77.8% 51.2%	Yes
**POSITIVELY CONTROLLED TRIALS – NOT TESTING A NON-INFERIORITY HYPOTHESIS**			
Wraight (42)	Only assessed if CC achieved; Etiology absent from all 3 post-treatment samples	75.0% 64.3%	No
McDougall (43)	Etiology absent in 21 d sample	77.0% 77.0%	No
Taponen et al. (44)	Etiology absent in 26 d sample	73.2% 78.7%	No
Sérieys et al. (45)	Etiology absent at both 17 & 22 d	54.3% 45.9%	No
Taponen et al. (46)	Etiology absent in 21- 28 d samples	56.1% 79.1% 33.3% 33.3%	Yes
Wenz et al. (33)	Culture negative 7 d after leaving hospital	27.0% 45.0% 33.0% 52.0%	No
Bradley and Green (47)	Etiology absent in both 16 and 25 d	Not reported	No
Swinkels et al. (48)	Etiology absent in both 14 and 21 d samples	72.0% 79.0%	No
Kalmus et al. (49)	PCR negative at d 21 & 28	54.1% 55.7%	No
Truchetti et al. (50)	Etiology negative at d 7, 14, & 21	32.0% 61.0%	Yes
Cortinhas et al. (51)	Etiology absent at 14 or 21 d	79.0% 76.0%	No
Viveros et al. (52)	Etiology absent in 7, 14 & 21 d samples	90.0% 95.1% 88.9% 83.3%	No
McDougall et al. (53)	Etiology absent in both 14 & 21 d	81.2% 83.8%	No
**POSITIVELY CONTROLLED TRIALS – TESTING A NON-INFERIORITY OR “EQUIVALENCY” HYPOTHESIS**			
McDougall et al. (54)	Etiology absent at 21 d	84.0% 81.0% 85.0%	yes
McDougall et al. (21)	Etiology absent in both 14 and 21 d samples	73.3% 72.0%	No
Schukken et al. (55)	Etiology absent at d 10 and 17	61.0% 73.0%	No
Vasquez et al. (56)	Etiology absent at 14, 21 d samples	72.0% 67.0%	No
Bryan et al. (57)	Only assessed if CC achieved; Etiology absent at d 9,16, & 23	57.2% 65.7%	No
Tomazi et al. (58)	Etiology absent in 14 and 21 d samples	68.0% 73.0%	No

a *Proportion of BC are listed in same order of treatments defined in Table 1*.

Bacteriological cures result from the combined effect of the host immune response (spontaneous cure) and the effect of treatment (treatment cure) and the value of antimicrobial therapy is greatest for pathogens that have a low rate of spontaneous cure and high rate of treatment cure (such as IMI caused by *S. agalactiae*). Among mastitis pathogens, expected rates of spontaneous bacteriological cure vary widely. The greatest contrast is between expectations of spontaneous bacteriological cure of IMI caused by *S. aureus* (close to zero) and *Escherichia coli* (about 90%) (25, 26). Limited efficacy of antibiotic therapy is well-documented for IMI caused by *S. aureus* (38, 64) and some pathogens lack targets for antimicrobial therapy (e.g., yeast, *Prototheca bovis, Mycoplasma* spp. and others) and are considered to be intrinsically resistant to all approved antimicrobials. It is important to reiterate, that even with highly efficacious drugs the benefit of antimicrobial therapy is only for the cases that do not achieve spontaneous bacteriological cure; thus, the marginal value of antibiotic therapy decreases for cases caused by *E. coli* or other pathogens with high rates of spontaneous cure.

Among the 23 trials that evaluated bacteriological cure, statistically significant differences among treatments were observed in only 4 trials while 16 reported no significant differences and 3 trials concluded the evaluated treatments were non-inferior (Table 3). Two of the trials that reported significant differences in BC enrolled only Gram-negative cases and compared IMM antimicrobial treatment to non-treated controls (25, 40). The distribution of pathogens in both studies included a considerable proportion of *Klebsiella* spp. which influenced overall BC of the combined groups. Fuenzalida and Ruegg (25) identified an interaction of pathogen by treatment group and reported BC of 18% (non-treated *K. pneumoniae*), 74% (treated *K. pneumoniae*), 97% (non-treated *E. coli*) and 99% (treated *E. coli*). Schukken et al. (40) reported significant differences in BC for cases caused by both *E. coli* and *Klebsiella* spp. but this study is unique in reporting exceptionally low spontaneous cure rates of *E. coli* cases, which the authors attributed to persistent infections. In spite of finding significant differences in BC, neither of these studies reported biologically important differences in important clinical outcomes. Taponen et al. (46) reported BC of clinical mastitis caused by *S. aureus* that were either β-lactamase negative or positive and were treated with either systemic penicillin & an IMM combination product containing penicillin & neomycin or received systemic penicillin alone. While a significant difference was identified among treatments, BC was dramatically decreased for β-lactamase positive organisms. Truchetti et al. (50) compared shorter (2d) vs. longer (5d) therapy using IMM ceftiofur and reported a significant difference in BC but no differences in any clinical outcomes. Over 30% of cases enrolled in this study were culture-negative and 26% were caused by *S. aureus* thus the impact of 3d difference in therapy was likely biologically irrelevant. In general, no clear relationship between BC and important clinical outcomes (such as new IMI, clinical cure, recurrence, SCC, milk yield or culling) were apparent (Table 4). Thus, while achieving BC is the goal of antimicrobial therapy, the finding of differences in BC in research trials does not appear to be predictive of differences in clinical outcomes.

**Table 4:** Statistical significance of other study outcomes categorized by significance of bacteriological cure in 23 clinical trials that evaluated bacteriological cure.

**Bact. cure**	**New IMI**		**Clin. cure**		**SCC**		**Recur**		**Milk yield**	
Result	*N*^a^	Sig^b^	*N*	Sig	*N*	Sig	*N*	Sig	*N*	Sig
Sig. difference	1	0	4	1	3	0	1	0	2	0
(*n* = 4 studies)										
No sig. diff. or non-inferior	3	0	19	7	10	2	2	1	4	1
(*n* = 19 studies)										

a *Number of studies evaluating the outcome*;

b *number of studies reporting significant difference among treatment groups in that outcome*.

New Intramammary infections are typically defined based on recovery of a different pathogen in follow-up milk samples but in the study that enrolled culture negative cases (22), NIMI was defined as recovery of any pathogen in the 14 and 21 d follow-up samples. While this outcome was not significant in any trials, recovery of both the etiological agent and new pathogens from follow-up samples after treatment is usually greater at earlier sampling periods (22, 25) as compared to samples collected after 14 d and in some instances may reflect differences among pathogens in duration of time to achieve both spontaneous and treatment clearance.

## Clinical Outcomes

### Clinical Cure (CC)

Almost all studies (*n* = 24; Table 5) evaluated “clinical cure” but the definition of this outcome varies enormously. Most researchers (67%) defined CC based on observations that the milk and/or the mammary gland returned to normal appearance, but the day of observation varied from 2 to 28 days after treatment and some relied on single observations, while other researchers required multiple observations. Of studies that defined CC based on observation of clinical signs, the proportion achieving CC was least (CC<15%) for studies that performed observations very early and evaluated systemic therapies (27, 41). For CC estimated based on visual observations after day 3, values of CC ranged from 25% to 98% with a median value of 81%. Other definitions of CC included retreatment within a defined time period (4 studies), use of scoring systems (2 studies) or a combination of methods (2 studies). Of 7 studies that reported significant differences in CC among treatments, 4 defined CC based on retreatment, 2 used comparison of defined scoring systems and 1 evaluated CC within 4 days of treatment. While achieving CC is the practical goal on farms, this outcome is not useful to determine effectiveness of antimicrobial therapy. In most cases of clinical mastitis, inflammation is self-limiting and regardless of BC, and for the majority of cases, milk will return to normal appearance by day 7 (30). There is almost no evidence that selection of an antimicrobial has a significant impact on this outcome and CC should not be used to make decisions about treatment efficacy.

**Table 5:** Clinical cure definition and outcomes for 24 studies reporting this outcome.

**Study**	**Clinical cure definition**	**% Clinical cure^**a**^**	**Sig. treatment or pathogen effects**
**NEGATIVELY CONTROLLED RANDOMIZED CLINICAL TRIALS**			
Roberson et al. (39)	Normal milk for 3 d or 2 weeks without recur assessed on d 7 & 36	57% Rx1 25% Rx2 53% Rx3 64% Con	No treatment effect Large pathogen effect
Suojala et al. (27)	Absence of signs and normal milk at d2 and/or d21	Day 2: 8% Rx1 & 20% Con Day 21: 47% Rx2 & 57% Con	Sig. Treatment effect at d2 but not d21
Schukken et al. (40)	<50% of original case severity score at 7 & 14 d	54% Rx1 46% Con	Sig. treatment & pathogen effects
Persson et al. (41)	Absence of clinical signs and normal milk on d 3	21% Rx1 11% Con	Not reported
Fuenzalida and Ruegg (22)	Return to normal milk for 2 consecutive d within first 10 d after treatment	86% Rx1 92% Con	No treatment effects
Fuenzalida and Ruegg (25)	Return to normal milk for 2 consecutive d within first 10 d after treatment	92% Rx1 98% Rx2 90% Con	No treatment or pathogen effects
**POSITIVELY CONTROLLED TRIALS - NOT TESTING A NON-INFERIORITY HYPOTHESIS**			
Erskine et al. (34)	Additional treatment required within 48 h of initial therapy	84% Rx1 77%Rx2	Sig. treatment effect only for coliform cases
Wraight (42)	Normal milk at end of milk withholding	83% Rx1 81% Rx2	No treatment effect Significant effect of pathogen
McDougall (43)	Re-treatment within 21 d of enrollment	16% Rx1 5% Rx2 (% retreated)	Significant treatment effect
Taponen et al. (44)	No systemic or local signs evident by 3–4 weeks post-treatment	73% Rx1 79% Rx2	No treatment effect
Sérieys et al. (45)	Return to normal milk & udder at 3, 8, 17, & 22 d	>80% for all groups at all periods	No treatment effect
Sérieys et al. (45)	No systemic or local signs evident by 3–4 weeks post-treatment	75% Rx1 74% Rx2	No treatment effect CNS CC higher
Swinkels et al. (48)	Severity grade 0 at d 1.5, 5, 14, 21	60% Rx1 82% Rx2	Significant treatment effect
Kalmus et al. (49)	Normal milk and gland by 21 to 28 d	80% Rx1 75% Rx2	No treatment effect
Truchetti et al. (50)	Normal milk 21 d after last treatment	89% Rx1 89% Rx2	No treatment effect
Cortinhas et al. (51)	Normal Milk & Glands on d 4, 14, and 21 d	79% Rx1 74% Rx2	No treatment effect No pathogen effect
Viveros et al. (52)	Absence of signs 4 d after 1st treatment	95% Rx1 96% Rx2 68% Rx3 58% Rx4	Significant effect of Treatment
McDougall et al. (53)	Visually abnormal milk at 14 & 21 d	82% Rx1 81% Rx2	No treatment effect
**POSITIVELY CONTROLLED TRIALS – TESTING A NON-INFERIORITY OR “EQUIVALENCY” HYPOTHESIS**			
McDougall et al. (54)	No new treatment within 30 d	86% Rx1 80% Rx2 84% Rx3	Significant effect of treatment
McDougall et al. (21)	No re-treatment within 21 d	72% Rx1 87% Rx2	Significant effect of treatment CC sig worse in *S. aureus* CC sig better in no growth
Schukken et al. (55)	Normal milk and gland at 10 and 17 d	62% Rx1 62% Rx2	No significant Rx Effect Significant pathogen effects
Vasquez et al. (56)	Normal milk and udder 2 to 5 d	64% Rx1 68% Rx2	No Treatment effect
Bryan et al. (57)	Return to normal milk and no further RX up to day 23	80% Rx1 80% Rx2	No significant treatment or pathogen effects
Tomazi et al. (58)	Milk and gland normal 48 h after last treatment	88% Rx1 94% Rx2	No No

a *Proportions of clinical cure are listed in same order of treatments defined in Table 1*.

### Post-treatment SCC

Similar to CC, a variety of definitions and sampling periods were used to assess SCC responses in the 14 studies that included this outcome (Table 6). Seven studies each assessed SCC at the quarter or composite level and dilution of healthy milk from unaffected quarters likely influenced results of studies that used composite milk samples. Sampling periods ranged from 7 to 90 days after treatment, and all studies that assessed SCC at multiple periods reported a gradual decline in SCC as time passed. While some researchers compared linear scores, other researchers compared the proportion of samples that were defined as “healthy” based on a threshold or either 200,000 or 250,000 cells/mL. Of the 14 studies that included this outcome, only 3 reported significant differences in their measure of SCC. One researcher used the California Mastitis Test on quarter milk samples collected at day 36 post-treatment and reported significantly fewer quarters below “trace score” for quarters that did not receive IMM treatment but received frequent milking (39). Interestingly, no difference was seen in non-treated control quarters that were not frequently milked. In another study, fewer quarters that received IMM antibiotics (as compared to systemic) achieved SCC <250,000 cells/mL by 22 days post-treatment (45) and a 3rd study reported significantly lower SCC at days 7 and 14 but those effects were not significant by day 22 (52). When assessing SCC responses, it is important to recognize that quarter-level measurements will more accurately reflect ongoing inflammation that may indicate persistent IMI. When BC is achieved SCC will gradually decline and the speed of return to a “healthy” level is influenced by etiology. Assessment of SCC responses should be performed at the quarter level and should continue for at least 21 days. Somatic cell count responses are a practical outcome that can be used as an indicator of treatment success on farms, but a gradual (rather than immediate) decline should be expected. When using composite milk samples, a lower threshold (<150,000 cells/mL) may help prevent misclassification of on-going subclinical infections that can result after failure to cure clinical cases.

**Table 6:** SCC definitions and outcomes for 14 studies reporting this outcome.

**Study**	**SCC outcome definition**	**Quarter or composite**	**Response valuation**	**Sig. treatment or pathogen effects**
**NEGATIVELY CONTROLLED RANDOMIZED CLINICAL TRIALS**				
Roberson et al. (39)	% quarters with CMT < trace at day 36	Quarter	54% Rx1 21% Rx2 43% Rx3 44% Con	SIG difference between Rx1 and Rx2
Schukken et al. (40)	Linear SCS value at 1st & 2nd post-treatment test day	Composite	5.5 Rx1 5.4 Con	No treatment effect
Persson et al. (41)	Comparison of median SCC at monthly post-treatment test days for 6 months (month 6 shown)	Composite	58,000 cells/mL 123,000 cells/mL	No treatment effect
Fuenzalida and Ruegg (22)	Post-treatment SCC weekly until 90 DIM	Quarter	5.5 Rx1 5.4 Con	No treatment effects
Fuenzalida and Ruegg (25)	Post-treatment SCC weekly until 90 DIM	Quarter	6.3 Rx1 6.0 Rx2 6.1 Con	No treatment effects
**POSITIVELY CONTROLLED TRIALS - NOT TESTING A NON-INFERIORITY HYPOTHESIS**				
McDougall (43)	Test day SCC values	Composite	Values not shown	No treatment effects
Sérieys et al. (45)	SCC at days 8, 17, & 22 post-treatment; Also compared % of quarter SCC >250,000 cells/mL (day 22 data for % <250,000 shown)	Composite	70% Rx1 57% Rx2	SCC of quarters that received IMM were significantly greater
Swinkels et al. (48)	SCC collected between days 21–27 post-treatment; compared median values and % of quarters with SCC <200,000 cells/mL	Quarter	24% Rx1 31% Rx2	No treatment effects
Kalmus et al. (49)	SCC collected monthly for 3 months post-treatment; also reported % below 200,000 cells/mL (month 3 data shown)	Composite	43% Rx1 51% Rx2	No treatment effect
Viveros et al. (52)	SCC at days 7, 14 & 21 post-treatment; only assessed on clinically cured cows	Quarter	Values shown only in figure	Significant effect of treatment at days 7 & 14 but not day 21
McDougall et al. (53)	Linear SCS at days 14 & 21 post-treatment (day 21 data shown)	Quarter	6.4 Rx1 6.3 Rx2	No treatment effect
**POSITIVELY CONTROLLED TRIALS – TESTING A NON-INFERIORITY OR “EQUIVALENCY” HYPOTHESIS**				
McDougall et al. (21)	Linear SCS at months 1–3 post-treatment	Composite	4.5 Rx1 4.4 Rx2	No treatment effect
Vasquez et al. (56)	Linear SCS at 1st month post-treatment	Composite	3.1 Rx1 3.4 Rx2	No Treatment effect
Tomazi et al. (58)	Proportion of linear SCS <4.0 at day 21 post-treatment	Quarter	29% Rx1 28% Rx2	No Treatment effect

### Recurrence, Milk Yield, and Retention Within the Herd

A few studies have evaluated other important clinical outcomes. Only 4 studies reported recurrence as a distinct outcome (22, 25, 33, 43) but several included recurrence in their definition of clinical cure (or “clinical failure”) (53, 54). Like other outcomes, recurrence can be defined at either the quarter or cow level, but when used to assess treatment this outcome should always be assessed relative to the affected quarter. Recurrence ranged from about 5–30% and was strongly influenced by additional risk factors such as parity (older cows are at greater risk of recurrence), etiology (culture positive are at greater risk as compared to culture negative), and increased milk yield. While two studies reported significant differences in recurrence based on treatment, this outcome is influenced by many other factors and should be interpreted cautiously. Post-treatment milk yield is an obviously important outcome that requires a prolonged follow-up period and has been evaluated infrequently in mastitis trials (22, 25, 39, 40, 43, 54). Of the 6 studies evaluating this outcome, 4 included a non-treated control group and the only significant finding was one study that reported non-treated control animals had the greatest post-treatment milk yield (39). Similarly, a significant difference in retention (or culling) after treatment was reported in only 1 of 8 studies that evaluated this outcome (22, 25, 27, 33, 40, 49, 55, 65). Culling is a very difficult outcome to assess as it is influenced by many factors including non-blinded studies that allow farmers to remove cows without a withholding period if they have not received antimicrobial therapy. While all of these outcomes are relevant and useful for dairy farmers, there is insufficient evidence to suggest that they are influenced by choices made about mastitis treatment.

## Conclusions and Clinical Implications

Over the last 20 years, very few mastitis trials have been conducted to differentiate among mastitis treatment protocols and the inclusion of multiple etiologies and culture negative cases in efficacy studies have resulted in little ability to differentiate among treatments. Few studies have been conducted that evaluated antimicrobial therapies approved to treat clinical mastitis in N. America. With rare exceptions, researchers have not reported significant differences in most microbiological or clinical outcomes and non-inferiority trials have not concluded that there are differences among products. There is no-evidence that IMM products approved for treatment of clinical mastitis caused by Gram-positive organisms vary in efficacy and other characteristics of approved products (dosing interval, withholding period, price etc.) can be used to make treatment decisions. When possible, etiology should be determined before treatment, the probability of spontaneous cure should be considered and the spectrum of antimicrobial activity of the approved product should be appropriate for the etiological agent. Research has demonstrated that cases of mastitis that are culture-negative at detection or are caused by *E. coli* rarely benefit from antimicrobial therapy and use of antimicrobials to treat these cases should be considered on an individual case basis. Associations between BC and clinical outcomes are very weak, and resolution of inflammation (duration of abnormal milk) is not a reliable indicator of therapeutic efficacy. Among potential indicators that can be used clinically, evaluation of continued decline in quarter-level SCC appears to be the most reliable indicator of success.

## Author Contributions

The author confirms being the sole contributor of this work and has approved it for publication.

## Conflict of Interest

The author declares that the research was conducted in the absence of any commercial or financial relationships that could be construed as a potential conflict of interest.
